# DNA methylation dynamics at imprinted genes during bovine pre-implantation embryo development

**DOI:** 10.1186/s12861-015-0060-2

**Published:** 2015-03-10

**Authors:** Alan M O’Doherty, David A Magee, Lynee C O’Shea, Niamh Forde, Marijke E Beltman, Solomon Mamo, Trudee Fair

**Affiliations:** School of Agriculture and Food Science, University College Dublin, Belfield, Dublin, Ireland; School of Medicine and Medical Sciences, University College Dublin, Belfield, Dublin 4, Ireland; College of Agriculture, Health and Natural Resources, Animal Science, University of Connecticut, Connecticut, USA

**Keywords:** Embryo, DNA methylation, Genomic imprinting, Bovine, Preimplantation embryos, Epigenetic reprogramming, DNA methyltransferases

## Abstract

**Background:**

In mammals, maternal differentially methylated regions (DMRs) acquire DNA methylation during the postnatal growth stage of oogenesis, with paternal DMRs acquiring DNA methylation in the perinatal prospermatagonia. Following fusion of the male and female gametes, it is widely accepted that murine DNA methylation marks at the DMRs of imprinted genes are stable through embryogenesis and early development, until they are reprogrammed in primordial germ cells. However, the DNA methylation dynamics at DMRs of bovine imprinted genes during early stages of development remains largely unknown. The objective of this investigation was to analyse the methylation dynamics at imprinted gene DMRs during bovine embryo development, from blastocyst stage until implantation.

**Results:**

To this end, pyrosequencing technology was used to quantify DNA methylation at DMR-associated CpG dinucleotides of six imprinted bovine genes (*SNRPN*, *MEST*, *IGF2R, PLAGL1*, *PEG10* and *H19*) using bisulfite-modified genomic DNA isolated from individual blastocysts (Day 7); ovoid embryos (Day 14); filamentous embryos (Day 17) and implanting conceptuses (Day 25). For all genes, the degree of DNA methylation was most variable in Day 7 blastocysts compared to later developmental stages (*P* < 0.05). Furthermore, mining of RNA-seq transcriptomic data and western blot analysis revealed a specific window of expression of DNA methylation machinery genes (including *DNMT3A*, *DNMT3B*, *TRIM28*/*KAP1* and *DNMT1*) and proteins (DNMT3A, DNMT3A2 and DNMT3B) by bovine embryos coincident with imprint stabilization.

**Conclusion:**

The findings of this study suggest that the DNA methylation status of bovine DMRs might be variable during the early stages of embryonic development, possibly requiring an active period of imprint stabilization.

**Electronic supplementary material:**

The online version of this article (doi:10.1186/s12861-015-0060-2) contains supplementary material, which is available to authorized users.

## Background

The epigenetic process of genomic imprinting enables parent-of-origin expression of a cohort of mammalian genes [1]. Imprinted genes have been shown to play a pivotal role in embryonic growth, development, placental function and postnatal behaviour and metabolism [2-5]. The distinctive monoallelic expression of imprinted genes is facilitated through asymmetrical epigenetic marks on either the maternal or paternal allele. Generally, imprinted genes are arranged in clusters containing differentially marked, CpG rich domains, known as imprint control regions (ICRs) and/or differentially methylated regions (DMRs); the most extensively studied of these marks is DNA methylation [6]. Mammalian DNA methylation patterns required for genomic imprinting are subject to periods of dynamic reprogramming during development and are established at different developmental time points, depending on which germline they are transmitted through. Paternal DMRs acquire their methylation marks in the prospermatagonia, with completion occurring prior to birth [7,8]; while DNA methylation marks at maternal DMRs are established postnatally in the growing oocyte [9-11].

Following fertilization, a global cascade of DNA demethylation is evident in the preimplantation embryos of a number of mammalian species including mouse, rat and cattle [12,13]. This demethylation event occurs actively on the paternal genome in one cell embryos [14,15] and passively on the maternal genome following each cell division from the two cell stage until blastocyst in mouse [16] and up to the 8-cell stage in bovine [12]. Interestingly, studies in mice have shown that the DNA methylation landscape at the DMR of the imprinted *H19* gene is resistant to these pan-genomic demethylation events [17,18]. Although information is available in mice [19-21] and there is some information on non-imprinted and imprinted genes in bovine embryos [22,23], the fate of maternally methylated imprinted gene DMRs remains largely undetermined during the early stages of bovine embryogenesis, especially so between blastocyst stage and implantation. Indeed, it has been suggested that murine DNA methylation imprints may be dynamic in the early embryo [24]. However, this study demonstrated that regions around maternally methylated germ line DMRs may undergo some changes, leaving a core DMR methylated. Extensive work has been carried out to elucidate which genes are involved with establishing and maintaining DNA methylation marks, and it is now widely accepted that the DNA methyltransferase genes (*Dnmt3a*, *Dnmt3b* and *Dnmt3L*) are responsible for establishing DNA methylation imprints in the male and female germline [25]; with *Dnmt1* and *Uhrf1* being primarily implicated with maintenance of methylation marks following DNA replication [26-28]. Gene targeting studies in mice have demonstrated that the methyltransferases Dnmt3a, Dnmt3b and Dnmt1 are indispensable for embryonic survival [29,30]. With regards to *Dnmt3L*, transgenic mice carrying homozygous null mutations are viable [31]; however, *Dnmt3L*^*-/-*^ male mice are infertile due to reactivation of long interspersed elements (LINE-1) and retrotransposons as well as meiotic catastrophe [32,33], while females fail to deliver viable pups as a result of hypomethylation at imprinted loci [34]. More recently, expression of *Trim28*/*Kap1* and *Zfp57* have been associated with the regulation of epigenetic stability in mouse oocytes and embryos [35,36]. In addition to establishing and maintaining DNA methylation marks, evidence suggests that the Ten-eleven translocation methylcytosine dioxygenase (TET) family members play a central role in active demethylation [37].

In cattle, developmental epigenetic research has primarily focused on cloned bovine embryos, due to the low survival rates of embryos produced by somatic cell nuclear transfer (SCNT). A high degree of SCNT embryonic loss occurs during the preimplantation period [38] with further loss and morphological anomalies (enlarged placentomes, enlarged umbilical cords and large offspring syndrome) being observed in embryos that successfully implant and progress through gestation [39-41]. It has been suggested that this low survivability (5 - 8% development to term) [42], may be due to erroneous epigenetic reprogramming [43,44], such as aberrant DNA methylation patterns observed at the imprinted *SNRPN* locus in Day 17 SCNT embryos [45]. Monoallelic expression and DNA methylation patterns of imprinted genes associated with the human epigenetic disorder, Beckwith-Wiedemann syndrome, have also been shown to be conserved in bovine Day 65 concepti [46]. The role of DNA methylation programming has also been highlighted in studies involving *DNMT1* siRNA-based knockdown experiments during *in vitro* development of bovine SCNT embryos. These investigations suggest that a significant increase in development to blastocyst was due to enhanced reprogramming efficiency elicited by DNA demethylation in *DNMT1* knockdown embryos [47]. In a recent study using fluorescent labelling techniques, Dobbs and colleagues revealed global methylation patterns in pre-implantation bovine embryos [48]. Results from this study showed that methylation patterns significantly differ between male and female embryos and that, in the blastocyst, the inner cell (ICM) mass is less methylated when compared to the trophectoderm (TE), confirming previous findings by Hou et al. [49]. However, in contrast to the investigations carried out on cloned embryos, non-manipulated *in vivo* derived embryos have received limited attention, particularly prior to Day 17.

In the current study, we investigated the DNA methylation profiles at DMRs of six bovine imprinted genes (*SNRPN*, *MEST*, *IGF2R, PLAGL1*, *PEG10* and *H19*) in *in vivo* derived embryos at several stages of development, including blastocyst and peri-implanting conceptuses. In addition, the abundance of RNA transcripts, and proteins, known to be associated with the establishment and maintenance of DNA methylation imprints were determined in embryos at parallel stages of development.

## Methods

### Ethical approval

All experimental procedures involving animals were licensed by the Department of Health and Children, Ireland, in accordance with the Cruelty to Animals Act, 1897, and the European Community Directive 86/609/EC. All procedures were sanctioned by the University College Dublin, Ireland Animals Research Ethics Committee. All animals were processed in a commercial abattoir.

### Animal synchronization and embryo collection

Collection of *in vivo* derived bovine embryos was performed using a previously described synchronization protocol [50,51]. Briefly, crossbred beef heifers (approximately 18-24 months old) were synchronized using an 8-Day CIDR treatment (Controlled Internal Drug Release device, 1.36 g progesterone; Pfizer Animal Health Worldwide) with administration of a prostaglandin PGF2α analogue (Estrumate MSD Animal Health, containing 0.5 mg cloprostenol) injection one Day prior to removal of the CIDR. Animals were checked for estrus four times daily, from 36 h following PGF2α injection. Animals that were in standing estrus between 36-60 h were inseminated using frozen thawed semen. Inseminated animals were slaughtered and embryos recovered from reproductive tracts on Days 7 (*n* = 10), 14 (*n* = 8), 17 (*n* = 10) and 25 (*n* = 8).

### DNA modification

Isolation and bisulfite modification of DNA was carried out using the EZ methylation direct method (Zymo Research) according to manufacturer’s guidelines. Firstly, proteinase k digestion was performed in a 20 μl reaction volume, containing 1 μg/μl proteinase K and 1× M-digestion buffer, at 50°C overnight. Sample input for the digests were as follows; individual Day 7 or 2 μl (from a total volume equal to 6 μl) of disaggregated Day 14, Day 17 embryonic disc and Day 25 embryo proper. For trophectoderm (Day 17) and extraembryonic (Day 25) samples a 2 ul aliquot was used. Following this, 130 μl of fresh CT conversion reagent (sodium bisulfite conversion solution) was added directly to the digests which were then incubated at 98°C for 8 min and 64°C for 8 h. Bisulfite DNA clean-up was carried out by adding the CT conversion reactions to Zymo-Spin columns pre-loaded with 600 μl M-binding buffer, centrifugation at 13,000 rpm, washing with 100 μl M-wash buffer, desulphonation at room temperature for 25 min and a further two washes with 200 μl M-wash buffer. Samples were eluted in 42 μl elution buffer, warmed to 50°C to enhance recovery. Liver, kidney and heart samples were modified as in [11]. For experiments using limited starting amounts of DNA, the DNA was extracted using an AllPrep DNA/RNA Micro Kit (Qiagen) and quantified using a Qubit® dsDNA high sensitivity assay kit and a Qubit® 2.0 Fluorometer (LifeTechnologies).

### Bisulfite PCR

Bisulfite PCR reactions were carried out in 25 μl reactions using primers outlined in (Additional file 1: Table S2) Each PCR reaction contained 1 X PCR buffer (minus MgCl_2_), 0.2 μm forward and reverse primer, 2 mM MgCl_2_, 1.25 U Platinum *Taq* DNA polymerase and 6 μl bisulfite DNA template. Amplification conditions were as follows: initial denaturation; 95°C 3 min, followed by 40 cycles of 95°C denaturation for 30 sec, variable °C annealing for 30 sec and 72°C elongation for 30 sec, with a final elongation step at 72°C for 5 min. All reagents were supplied by Invitrogen Life Technologies™.

### Pyrosequencing

Pyrosequencing assays for DMRs at maternally imprinted genes were verified previously [11]. The *H19* assay was designed according to previously identified DMR [52] and verified in Additional file 2: Figure S1. Biotin labeled bisulfite PCR products, verified by agarose gel electrophoresis, were made up to 80 μl reaction volumes containing 2 μl streptavidin-coated Sepharose beads (GE Healthcare), 20 μl nuclease free H_2_0 and 40 ul binding buffer (Qiagen). Mixtures were agitated, to enable binding of biotin labeled strands to beads, by shaking at room temperature for 5 min. Template-bead complexes were immobilized to individual prongs on a Pyromark Q24 vacuum manifold (Qiagen) and subjected to a 10 sec ethanol wash (70% ethanol, Sigma); 15 sec denaturation step (PyroMark denaturation solution) and final 20 sec wash step in 1 X PyroMark wash buffer. The vacuum manifold was turned off and the bound template-bead complexes released into 25 μl primer mixes containing 0.3 μM sequencing primer and PyroMark annealing buffer. Internal bisulfite controls were included in each pyrosequencing assay performed. Only sequences that passed the internal control (>95% bisulfite conversion) were included in the analysis.

### Statistical analysis of methylation data

For this study, DMR methylation value was treated as a continuous variable. Pairwise group comparison of the variance in methylation for each gene was analysed using Levene’s homogeneity of variance test [53], which assumes data are not normally distributed. A *P*-value threshold of ≤ 0.05 was chosen to identify significant differences in the variance between groups. All analyses were performed using the Minitab version 16 software package (Minitab Ltd, Coventry, UK).

### Gene and protein expression analysis

The TPM values for each gene of interest were retrieved from a bovine embryo RNA-seq dataset [54]. The TPM values were log transformed and analyzed by the statistical package SAS (SAS Institute, Cary, NC). Analysis was performed using general linear model procedure (PROC GLM) with Day as the main effect. Effect of Day on embryo/conceptus gene expression were separated by Tukey’s test and a *P* value of < 0.05 was considered significant. Gene expression data are presented as the mean TPM ± SE. DNA methyltransferase protein analysis was carried out using whole Day 7 embryos or small fragments of Day 13 and 25 embryos essentially as described previously [11].

### Experimental model

**Figure 1:**
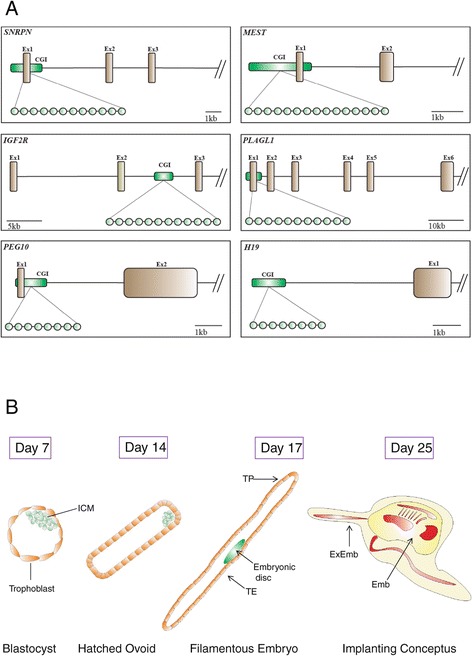
**Schematic representation of samples and imprinted genes analysed. (A)** Location of imprinted gene CpG islands (green box labelled GGI) and CpG dinucleotides covered by pyrosequencing analysis. Genes investigated were (clockwise from top left); *SNRPN*, *MEST*, *PLAGL1*, *H19*, *PEG10* and *IGF2R*. **(B)** *in vivo* embryos from non-stimulated, artificially inseminated heifers were isolated 7, 14, 17 and 25 Days post insemination and used for imprinted gene DNA methylation analysis. ICM = inner cell mass; TE = trophectoderm adjacent to embryonic disc; TP = trophectoderm peripheral; ExEmb = extraembryonic and Emb = embryonic.

The methylation profiles of six bovine genes (Figure 1A) known to undergo genomic imprinting were analysed [11,52] (see also http://www.geneimprint.com/site/genes-by-species.Bos+taurus). These included the maternally imprinted small nuclear ribonucleoprotein polypeptide N gene (*SNRPN*); mesoderm specific transcript homolog [mouse] gene (*MEST*); insulin-like growth factor 2 receptor gene (*IGF2R*); pleiomorphic adenoma gene-like 1 gene (*PLAGL1*); paternally expressed 10 gene (*PEG10*) and the paternally imprinted maternally expressed transcript gene (*H19*). The number of DMR-associated CpG dinucleotides analysed for each gene were as follows: *SNRPN* (*n* = 12, number of CpGs in island = 67); *MEST* (*n* = 15, number of CpGs in island = 119); *IGF2R* (*n* = 12, number of CpGs in island = 108); *PLAGL1* (*n* = 11, number of CpGs in island = 84); *PEG10* (*n* = 8, number of CpGs in island = 78) and *H19* (*n* = 8, number of CpGs in island = 45). The DNA methylation status of each individual CpG was determined by pyrosequencing, with the average methylation status of each gene (herein referred to as methylation value) calculated for single *in vivo* embryos at different stages of development (Additional file 3: Table S1).

Investigations performed using mice models [16] have demonstrated that DNA methylation at imprinted loci is resistant to early embryonic DNA methylation reprogramming events. Therefore, we hypothesised that a similar mechanism exists at bovine imprinted loci. To test this hypothesis, the methylation patterns at a panel of DMRs, were assayed in embryos (Figure 1B) across the pre- and peri-implantation axis: blastocyst (Day 7), hatched ovoid embryo (Day 14), filamentous embryo (Day 17) and implanting conceptus (Day 25). In addition, the methylation status of these DMRs was analysed in trophectoderm (Day 17) and extra embryonic (Day 25) tissues. For the Day 17 embryos two sections of the trophectoderm, embryonic disc adjacent (TE) and trophectoderm peripheral (TP), were analysed, as differences in morphology [55] and function [56] have previously been identified between regions adjacent to the embryonic disc and the periphery of the trophectoderm. A minimum of 4 individual embryos were assayed at each time point.

## Results

### DMR methylation during pre-implantation embryogenesis

**Figure 2:**
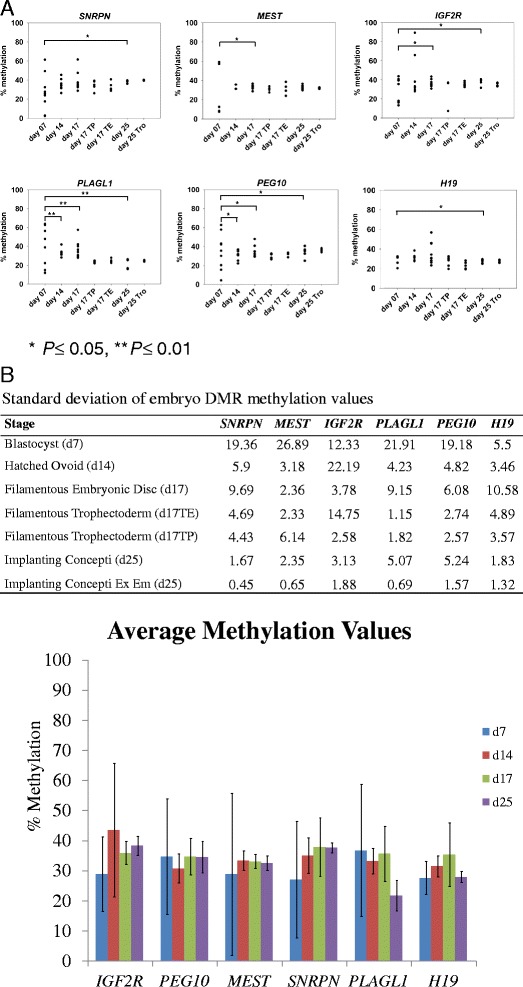
**Pyrosequencing analysis of DNA methylation at six bovine imprinted differentially methylated regions during early embryogenesis.** Bisulfite PCR and pyrosequencing was performed using genomic DNA isolated from embryos (*n* = 8 - 10) at four separate time points; blastocyst (Day 7), hatched ovoid embryos (Day 14), filamentous embryos (Day 17) and implanting conceptuses (Day 25). At Day 17, trophectoderm embryonic regions adjacent (TE) and peripheral to the embryonic disc (TP) were analysed. Trophectoderm samples at Day 25 were also included in the analysis. An overall trend of imprint stabilization can be observed for the six genes with increasing Days of embryonic development, post blastocyst. **(A)** Methylation values for *SNRPN*, *MEST*, *IGF2R*, *PLAGL1*, *PEG10* and *H19* at Day 7 had the most significant differences, when compared to Days 14, 17 or 25. **(B)** Top Panel; Standard deviations were calculated using individual methylation values (*n* = 4 - 9) for all genes in each group. Overall, standard deviations are greatest in group 1 (Day 7 blastocyst) with the smallest degree of variation occurring in the Day 25 implanting concepti (group 6) and Day 25 implanting concepti extra embryonic region. Bottom panel; Average methylation values, plotted with standard deviation values, for embryos isolated 7, 14, 17 and 25 Days post insemination (groups 1-3 and 6 from top panel).

Comparison across the different embryonic stages revealed that the greatest range in methylation values occurred at the Day 7 blastocyst stage (3-61% [*SNRPN*]; 7-59% [*MEST*]; 13-44% [*IGF2R*]; 12-64% [*PLAGL1*]; 7-59% [*PEG10*] and 20-32% [*H19*]), followed by the Day 14 hatched ovoid embryos (27-45% [*SNRPN*]; 31-36% [*MEST*]; 28-89% [*IGF2R*]; 31-42% [*PLAGL1*]; 22-37% [*PEG10*] and 21-31% [*H19*]). Notably, the range of methylation values for all six genes analysed were narrower at the Day 17 and Day 25 embryonic stages (Figure 2).

Previous studies have shown that methylation at DMRs of imprinted genes is stably maintained throughout early embryonic development [57]. For each gene, we compared the variances in sample group methylation across the development time course. For this, each developmental stage was considered a sample group and all possible pairwise group combinations were compared using the Levene’s test [53] for homogeneity of variance implemented in Minitab. This test does not assume an underlying normal distribution and is appropriate for comparing variances when sample sizes are small [58]. We hypothesised that stable inheritance and maintenance of methylation at imprinted loci across the pre-implantation developmental stages analysed will result in non-significant differences in the variance of methylation values between sample groups (Levene’s test *P* ≥ 0.05). In contrast, fluctuations in methylation patterns across the pre-implantation developmental will result in significant differences in the variance of methylation values across sample groups (Levene’s test *P* ≤ 0.05).

**Figure 3:**
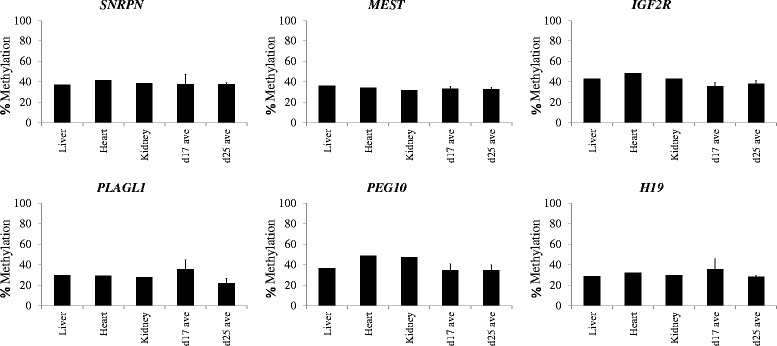
**Embryonic imprints at Day 17 and Day 25 methylation patterns resemble those observed in adult somatic tissues.** To investigate whether DNA methylation imprints remained stable, levels for each gene (*SNRPN*, *MEST*, *IGF2R*, *PLAGL1*, *PEG10* and *H19*) were analysed in several adult somatic tissues (*n* = 1 for each); Liver (Li), Heart (He) and Kidney (Kid) and compared to the average methylation values obtained at Day 17 and Day 25 (Additional file 3: Table S1), for each gene. The methylation values were, *SNRPN*; 37% (Li), 42% (He), 39% (Kid), 39% (Day 17) and 38% (Day 25), *MEST*; 36% (Li), 34% (He), 32% (Kid), 33% (Day 17) and 33% (Day 25), *IGF2R*; 43% (Li), 49% (He), 43% (Kid), 36% (Day 17) and 38% (Day 25), *PLAGL1*; 30% (Li), 29% (He), 28% (Kid), 36% (Day 17) and 22% (Day 25), *PEG10*; 37% (Li), 49% (He), 47% (Kid), 35% (Day 17) and 35% (Day 25) and *H19*; 29% (Li), 32% (He), 30% (Kid), 35% (Day 17) and 28% (Day 25).

Pairwise group comparisons revealed statistically significant differences (*P* ≤ 0.05) between the variance in methylation values between the Day 7 blastocysts relative to the later embryos for all genes. The variance of the *PLAGL1* and *PEG10* DMR methylation values at Day 7 was significantly greater to the variances observed for all three later developmental stages. In addition, the variance of the *IGF2R* methylation at Day 7 was significantly greater to that for the Day 17 and Day 25 embryos. Significantly greater variances were also observed for *MEST* (Day 7 versus Day 17), *H19* and *SNRPN* (both Day 7 versus Day 25). Notably, pairwise comparison of the variance in methylation values of the six gene-associated DMRs at the later developmental stages (Day 14, Day 17 and Day 25) revealed no statistically significant differences (*P* ≥ 0.05). Furthermore, Day 17 and Day 25 embryonic methylation values were similar to those of adult somatic samples, from heart, liver and kidney (Figure 3). To address the possibility that the observed variability in the blastocyst stage embryos could be due to technical, not biological, variation pyrosequencing analysis was performed using limited starting amounts of DNA- representative of the DNA content from a single (~1 ng DNA) or half (~500 pg DNA) a bovine blastocyst. Variation of DMR methylation between the two groups was assessed using the Levene’s test outlined above. No significant differences were observed between the two starting input amounts of DNA (Additional file 4: Figure S2A). However, further analysis of a subset of DMRs (*SNRPN*, *PEG10* and *H19*) using D7 methylation values and methylation values using limited starting amounts of embryonic and somatic (heart and liver) DNA samples revealed some significant differences for *SNRPN* and *PEG10* (Additional file 4: Figures S2B-C) and no differences for *H19* (Additional file 4: Figure S2D). When methylation was analysed using D17 methylation values (Additional file 3: Table S1) with low input (LI) D17 samples, with same cell equivalents of D7 samples, *SNRPN* and *PEG10* showed no significant differences (Additional file 4: Figure S2B (i) & S2C (i)). In a similar experiment using D25 samples *SNRPN* showed significant difference, while *PEG10* did not (Additional file 4: Figure S2B (ii) & S2C (ii)). Comparison of D7 methylation values (Additional file 3: Table S1) with LI somatic and LI embryonic methylation values demonstrated that D7 values were significantly different to LI somatic values and not to LI embryonic methylation values (Additional file 4: Figure S2B (iii) & S2C (iii)).

### Trophectoderm DMR methylation

As the elongating/filamentous embryo is comprised of embryonic disc and trophectoderm tissues, we analysed and compared the variance in methylation values for each gene-associated DMR for these tissues at the Day 17 developmental stage. Three sample types were included in this analysis—embryonic disc (ED), trophectoderm peripheral (TP) and trophectoderm adjacent to the embryonic disc (TE). Comparisons of variance in methylation values were performed for each gene between ED and TE, ED and TP, and TP and TE. With the exception of the comparison between the ED and TE tissues (*P* = 0.04) for *MEST*, there were no significant differences in the variance methylation observed (Figure 2A). Similarly comparison of variance in methylation values was carried out using data from Day 25 trophectoderm and Day 25 embryo samples. With the exception of *SNRPN* (*P* ≤ 0.05) no significant differences were observed.

### Methylation machinery abundance during bovine embryogenesis

**Figure 4:**
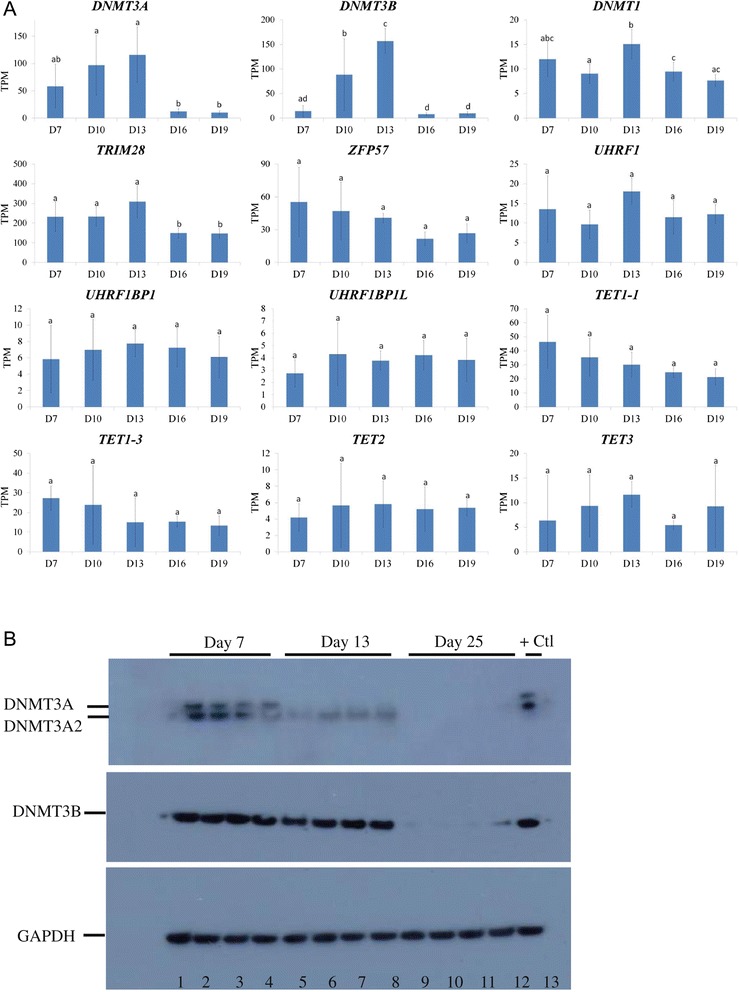
**Relative transcript abundance of** ***DNMT3A*** **,** ***DNMT3B*** **,** ***DNMT1*** **,** ***TET*** **family,** ***UHRF1*** **family,** ***TRIM28*** **/** ***KAP1*** **and** ***ZFP57*** **over five developmental stages. (A)** The TPM values for each gene of interest were retrieved from a bovine embryo RNA-seq dataset (43) generated from embryos (*n =* 5) at Day 7 (blastocyst), Day 10 (post hatching spherical embryo/conceptus), Day 13 (ovoid embryo/conceptus), Day 16 (filamentous embryo/coneptus) and Day 19 (filamentous embryo/conceptus; initiation of implantation). Bars with similar superscripts (abcd) are not significantly different (*P* < 0.05). A significant effect of embryonic stage on gene expression was observed for *DNMT3A*, *DNMT3B*, *DNMT1* and *TRIM28*/*KAP1*. Although a trend of decreasing transcript abundance is apparent for *TET1-1*, *TET1-3* and *ZFP57*, the decrease was not statistically significant. Transcripts per million (TPM). **(B)** Western blot analysis of the DNA methyltransferases; DNMT3A, DNMT3A2 and DNMT3B. All proteins were expressed in Day 7 blastocyst samples (*n* = 4) with DNMT3A and DNMT3B being expressed in all Day 13 embryos (*n* = 4). DNMT3 methyltransferase protein expression was not detectable in Day 25 conceptuses (*n* = 4). A positive control (+Ctl) of total protein extracted from oocytes (shown previously to express the DNMT3 family members) was separated alongside the embryo samples.

Analysis of global transcriptomic data generated by RNA-seq profiling of Day- 7, 10, 13, 16 and 19 bovine embryos [54], revealed an overall effect of embryonic stage on the RNA transcript abundance (transcripts per million, TPM) of genes associated with the establishment and maintenance of DNA methylation imprints, specifically *DNMT3A*, *DNMT3B*, *DNMT1*, and *TRIM28*/*KAP1* (*P* < 0.05, Figure 4A). These genes showed a significant temporal decrease in transcript copy number from Day 13 to 16 and Day 13 to 19 (*P* < 0.05). The expression of both *DNMT3A* and *DNMT3B* was also significantly reduced in Day 16 and 19 embryos compared to Day 10 embryos (*P* < 0.05). While, there was a trend for a decline in *ZFP57*, *TET1-1* and *TET1-3* mRNA abundances over time, this decrease was not statistically different. Transcripts for *TET* and *UHRF1* family members, although expressed across all stages, were not significantly different. *DNMT3L* expression was not detected above background levels (Additional file 5: Figure S4). In addition to analysis of RNA, we also performed western blot analysis to investigate the levels of the DNA methyltransferases; DNMT3A, DNMT3A2 and DNMT3B, using protein isolated from Day 7, 13 embryos and Day 25 conceptuses samples (Figure 4B). A similar pattern of expression was observed for these proteins that was also present in the RNA-seq analysis; demonstrating that both RNA and protein molecules are abundant during the period of DNA methylation imprint stabilisation.

## Discussion

The current study details the methylation dynamics at a panel of differentially methylated imprinted gene loci, during pre- and peri-implantation bovine development. Results generated from this investigation have yielded novel information regarding the stability of DNA methylation imprints in this early developmental window. We provide evidence suggesting that DMRs may be subjected to a period of reprogramming in cattle, during the ontogeny from bovine blastocyst to hatched ovoid embryo/conceptus.

Current understanding of the fate of gametic DNA methylation marks at imprinted genes following fertilization has come from studies in mice [17,18]. These investigations, for example, have provided robust information regarding the paternally transmitted *H19* DMR, demonstrating its stability during early embryonic development. Despite limited information regarding the early embryonic stability of maternally derived DNA methylation imprints, it is widely accepted that they follow a similar path to that of *H19* [16]. Here, we report for the first time a high level of variability in DNA methylation at the DMRs of imprinted genes in the bovine blastocyst, relative to levels detected at later stages of peri-implantation embryo development. This is similar to previous results reported in human that showed, using pyrosequencing, variability of DNA methylation at DMRs of imprinted genes in single blastocysts [59]. These findings suggest that there possibly could be a wave of imprint stabilisation post blastocyst stage, following which, embryonic DMR methylation values become comparable to those observed in adult somatic tissues.

This observed variability in methylation could, however, be due to a number of possible reasons. Firstly, it is possible that the variation in DMR methylation observed in the bovine blastocyst may be due, in part, to technical issues, such as amplification bias of maternally- or paternally-derived genome copies involving small starting amounts of template material. To address this, pyrosequencing of each DMR was carried out using small amounts of bisulfite-modified heart and liver DNA, equivalent to the DNA content of single (1 ng) and half a bovine blastocyst (0.5 ng). While the effect of preferential amplification of maternal or paternal alleles cannot be fully excluded in the current study, there was a lack of statistical differences in DMR methylation between the two limited DNA starting concentrations demonstrating reliability of the assays using small amounts of gDNA. However, conflicting results were observed when using low starting amounts of D17 or D25 DNA. No differences were observed at the *SNRPN*, *PEG10* or *H19* DMRs when analysing D17 methylation values, but *SNRPN* was significantly different between D25 samples, suggesting the possibility of technical variation. Secondly, it is possible that there is an overrepresentation of DNA from the ICM or the TE in the Day 7 samples. Previous investigations have demonstrated differential global methylation patterns between the ICM and TE in bovine blastocysts. However, the results are unclear with reports indicating that the TE is more methylated [48,49] and another showing higher methylation within the ICM [12]. Thirdly, the effect of hormonal treatment during animal synchronization on DMR methylation cannot be excluded as there is evidence to suggest that superovulation/ovarian stimulation regimes may interfere with methylation at the DMRs of imprinted genes in the murine blastocyst [60]. Fourthly, murine DMRs have been shown to be dynamic during murine pre-implantation development in their ability to expand, contract or shift [24]. To identify whether the DMRs interrogated in this study were behaving in a similar manner, and thus contributing to the observed variation, methylation of individual CpGs for each gene was assessed on an embryo by embryo basis in the Day 7 group (Additional file 6: Figure S3). These analyses revealed that variation was consistent across all the CpGs of each DMR, indicating that the methylation variability was unlikely to be due to a small number losing or gaining methylation. Finally, previous studies investigating methylation at imprinted genes (*H19* and *Igf2*) in embryonic germ cells, derived from either male or female primordial germ cells, identified that sex can contribute to differential methylation patterns at these loci [61-63]. Therefore, we cannot rule out that the sex of the embryos may be having an effect at DMRs at imprinted genes during the blastocyst stage.

Subsequent to the variability in methylation at D7, there was no difference in DMR methylation between trophectoderm regions and the embryo proper of implanting conceptuses, illustrating a pan-embryonic stability of DNA methylation at DMRs of imprinted gene. Considering the role of the trophectoderm in implantation and placenta formation [64], and given the role of genomic imprinting in placental function [65], these results are of particular interest. It has been previously proposed that normal function of the placenta may be impaired through alterations in the epigenetic landscape, such as those observed in cloned bovine placentas [66,67]. Here, we show that non-manipulated *in vivo* derived bovine embryos have a balanced methylation profile at DMRs of imprinted genes across the embryo proper and trophectoderm regions, suggesting that this may be the optimal imprinted gene methylation profile to support implantation and subsequent placental formation.

**Figure 5:**
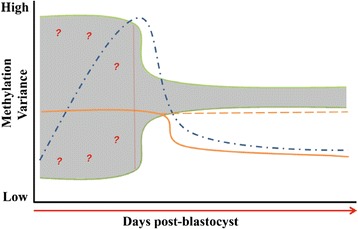
**Overview of DMR methylation at imprinted genes and expression of associated transcripts in pre-implantation bovine embryos.** Schematic representation of DNA methylation imprint dynamics following the blastocyst stage of bovine embryogenesis. Shaded grey area denotes the range of observed methylation values at imprinted loci during pre-implantation embryo development. Solid and dashed lines represents expression profiles of key genes associated with imprinted establishment and maintenance; blue dashed line *DNMT3A* and *DNMT3B*, solid orange line *DNMT1* and *TRIM28*/*KAP1* and solid orange-dashed orange line *ZFP57*, *UHRF1* family and *TET* family transcripts. Question marks to the left of the vertical dashed red line denote that technical variation cannot be completely excluded at this stage.

Targeted mining of bovine embryo global transcriptomic data [54] revealed a distinctive temporal RNA transcript profile for several key genes associated with establishing and maintaining imprints (*DNMT3A*, *DNMT3B*, *DNMT1*, *TRIM28*/*KAP1* and *ZFP57*). High levels of mRNA expression were observed during the period of greatest imprint instability, followed by a significant decrease in transcript abundance at later stages of development, in tandem with DNA methylation imprint stabilization. This pattern of expression was also observed for DNMT3A, DNMT3A2 and DNMT3B, complementing the gene expression profiles. In mice, *trim28*/*kap1* maternal knockouts present with severe phenotypic and epigenetic variability resulting in embryonic lethality [35], it has been proposed that an epigenetic complex formed by TRIM28/KAP1 and ZFP57 facilitate imprint maintenance/protection during the period of pre-implantation reprogramming [68-70]. In addition, the *de novo* and maintenance DNA methyltransferases, DNMT3A/B and DNMT1, were shown to interact with ZFP57 through its co-factor TRIM28/KAP1 to maintain methylation imprints in embryonic stem cells [36,69]. Taking all things in to consideration, it is likely that *TRIM28*/*KAP1* and *ZFP57,* along with the DNA methyltransferases *DNMT3A*, *DNMT3B* and *DNMT1* actively facilitate a window of DNA methylation reprogramming in bovine embryos post blastocyst development (Figure 5).

## Conclusion

In conclusion, this is the first comprehensive analysis of methylation patterns at maternally- and paternally- transmitted imprinted gene associated DMRs during early embryo development in cattle. In contrast to the murine model, our evidence suggests that bovine germline-acquired DNA methylation imprints may be susceptible to instability at the blastocyst stage and are subsequently stabilized during a period involving notable increases in the transcription of genes involved in the regulation of DNA methylation. However, variation in methylation in bovine blastocysts due to technical issues associated with limited amounts of DNA cannot be fully ruled out. Single nucleotide polymorphism analysis (SNP) to identify the ratio of maternal and paternal alleles, and therefore eliminate/confirm amplification bias, would be the most definitive approach to determine whether blastocyst methylation variability is biological or technical. Unfortunately this information was not available for this investigation, but it should be considered in future studies investigating imprinted gene methylation in samples with limited numbers of cells. Finally, our studies highlight the usefulness of larger mammalian species as models for investigating epigenetic regulation during embryo development.

## Availability of supporting data

All supporting data are included as additional files.

## Additional files

Additional file 1: Table S2. Bisulfite PCR pyrosequencing primers used in this study. Additional file 2: Figure S1. Verification of the bovine *H19* differentially methylated region. To confirm the methylation status of the *H19* DMR included in this investigation it was analysed using three techniques. Combined Bisulfite Restriction Analysis (A) and bisulfite sequencing show that this DMR is hypomethylated in the oocyte and hemi-methylated in liver. Pyrosequencing analysis of the same region confirmed these observations in oocytes and liver and also illustrated that *H19* is hypermethylated in sperm DNA. Additional file 3: Table S1. Individual methylation values for imprinted gene DMRs during bovine pre-implantation embryo development. Additional file 4: Figure S2A-B. DNA methylation analysis using limited starting amounts of genomic DNA as input for bisulfite conversions. Additional file 5: Figure S4.
*DNMT3L* mRNA expression during bovine embryonic development. Additional file 6: Figure S3. D7 individual CpG methylation analysis.
