# Supplementary material for: DNA methylation dynamics at imprinted genes during bovine pre-implantation embryo development
Source: BMC Dev Biol. 2015 Mar 10;15:13. doi: 10.1186/s12861-015-0060-2 (PMC4363183; doi:10.1186/s12861-015-0060-2)
Supplement: Additional file 1: Table S2. — Bisulfite PCR pyrosequencing primers used in this study. [file 12861_2015_60_MOESM1_ESM.pdf]

Table S2**.** Bisulfite PCR pyrosequencing primers used in this study.

Gene	Primer	Sequence 5'-3'	Annealing Temp (°C)	Accesion number
*SNRPN**	pyro fwd	TGA TTA TAG GGG TGG GGT AGA TAT TA	59	NM 001079797
	pyro rvs (biotin)	CAC CAC TCC CCA AAC TCC CCC TAA ACA TAC	59	
	pyro-seq	GGT TTT TTT GTT TGA GAG A		
*MEST**	pyro fwd	GGT ATT ATT GGG GTT TTT ATA TTG TGA T	55	NM 001083368
	pyro rvs (biotin)	CCA ACA CCT CCT AAA TCC CTA ACT A	55	
	pyro-seq	GGT GTT GGG AGT GGG TA		
*IGF2R**	pyro fwd	GAG AGG GTA GGA GTT AGA GTA GAA ATT TT	57	NM 174352
	pyro rvs (biotin)	CAC CTT ACT CAA AAC CTA CCA AC	57	
	pyro-seq	TTG GGT AGG GGG TTT		
*PLAGL1 /ZAC**	pyro fwd	GTT TGG GGA GGT ATT TAT AGG TTG AA	57	NM 001103289
	pyro rvs (biotin)	TCC CCA ACC AAA CCT CCT CCT AC	57	
	pyro-seq	AGG TTG AAT GAT AAA TGG		
*PEG10**	pyro fwd (biotin)	GTT TGG TAT AGG TGT GGG ATT T	56	NM 001172518
	pyro rvs	TCA AAA CCC TAA AAA CTT AAA TTC TC	56	
	pyro-seq	CCA CCC AAT TTT AAA ATA CAA CCA		
H19 †	pyro fwd (biotin)	TTT TGT GGA TTA TTG TGG TAT T	52	NR 003958
	pyro rvs	ATC TTA AAC TAA TCT CCC AAC CC	52	
	pyro-seq	CTA CAC ACC ACA AAA T		

Pyrosequencing assays for maternally imprinted gene differentially methylated regions (*) were described previously [11]. For *H19* (†), a pyrosequencing assay was designed in accordance to the genomic sequence described by Curchoe et al. [52].
