# Supplementary material for: DNA methylation dynamics at imprinted genes during bovine pre-implantation embryo development
Source: BMC Dev Biol. 2015 Mar 10;15:13. doi: 10.1186/s12861-015-0060-2 (PMC4363183; doi:10.1186/s12861-015-0060-2)
Supplement: Additional file 2: Figure S1. — Verification of the bovine H19 differentially methylated region. To confirm the methylation status of the H19 DMR included in this investigation it was analysed using three techniques. Combined Bisulfite Restriction Analysis (A) and bisulfite sequencing show that this DMR is hypomethylated in the oocyte and hemi-methylated in liver. Pyrosequencing analysis of the same region confirmed these observations in oocytes and liver and also illustrated that H19 is hypermethylated in sperm DNA. [file 12861_2015_60_MOESM2_ESM.pdf]

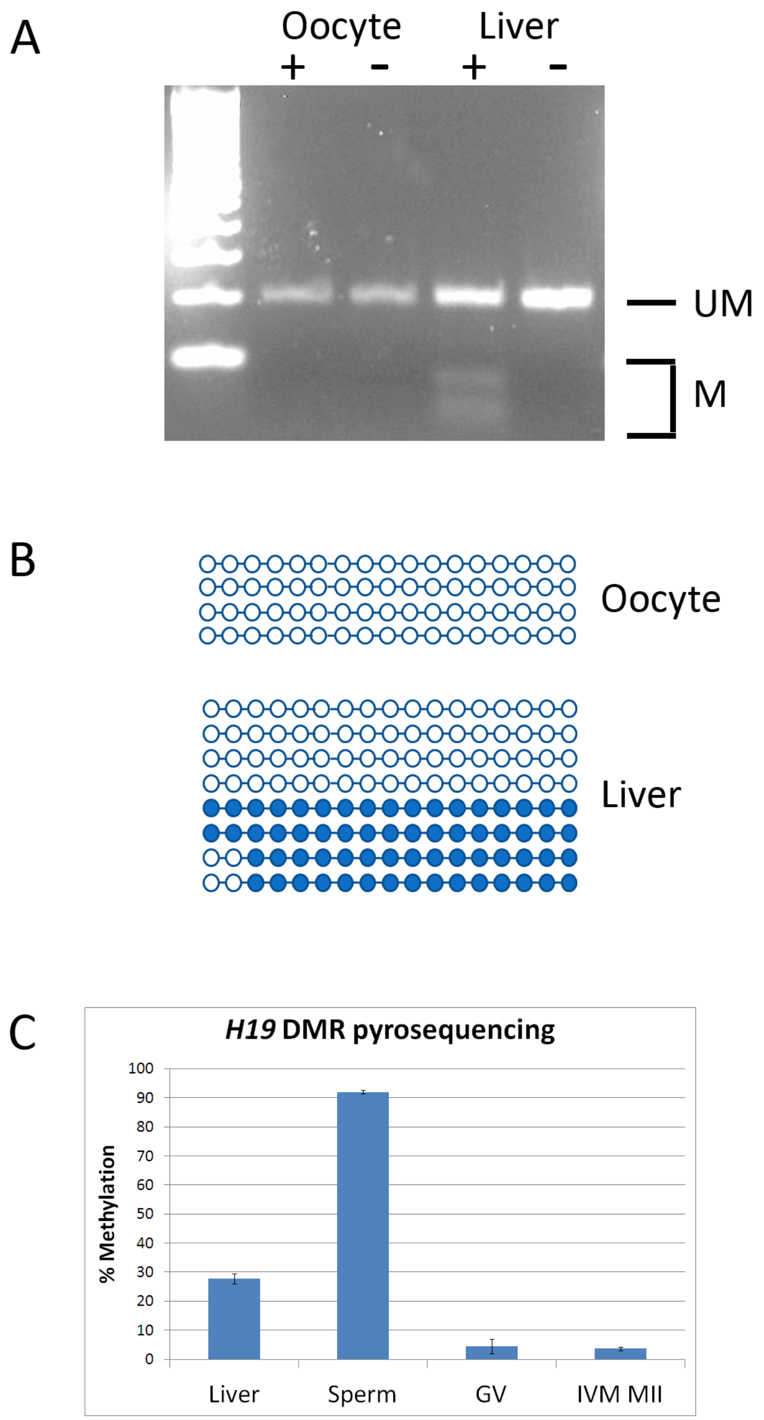
A
Oocyte
Liver
+
-
+
-
UM
M
B
Oocyte
Liver
C
H19 DMR pyrosequencing
% Methylation
100
90
80
70
60
50
40
30
20
10
0
Liver
Sperm
GV
IVM MII

Figure S1. Verification of the bovine *H19* differentially methylated region.

To confirm the methylation status of the *H19* DMR included in this investigation it was analysed using three techniques. Combined Bisulfite Restriction Analysis (A) and bisulfite sequencing show that this DMR is hypomethylated in the oocyte and hemi-methylated in liver. Pyrosequencing analysis of the same region confirmed these observations in oocytes and liver and also illustrated that *H19* is hypermethylated in sperm DNA.
