# Supplementary material for: DNA methylation dynamics at imprinted genes during bovine pre-implantation embryo development
Source: BMC Dev Biol. 2015 Mar 10;15:13. doi: 10.1186/s12861-015-0060-2 (PMC4363183; doi:10.1186/s12861-015-0060-2)
Supplement: Additional file 3: Table S1. — Individual methylation values for imprinted gene DMRs during bovine pre-implantation embryo development. [file 12861_2015_60_MOESM3_ESM.pdf]

Table S1. Individual methylation values for imprinted gene DMRs during bovine pre-implantation embryo development.

Sample	Group	*IGF2R*	*PEG10*	*MEST*	*SNRPN*	*PLAGL1*	*H19*
d7 1	1	40.8±5.6	62.8±12.3		27.7±7.8		31.4±6.0
d7 2	1	16.2±5.9	20.5±10.8		25.7±10.0	22.4±1.4	
d7 3	1	35.7±3.3			61.4±12.9	56.3±8.7	
d7 4	1	17.5±2.5	15.5±9.2		49.4±8.4	14.8±1.5	26.1±5.5
d7 5	1	17.9±3.2	30.3±5.0	7.2±2.2	32.4±10.3	11.8±1.4	
d7 6	1	13.3±7.2	35.8±16.4	8.1±2.9	17.5±10.6	12.0±3.2	
d7 7	1	43.8±15.2	57.8±7.7	59.1±5.7		62.7±20	
d7 8	1		4.5±1.6		2.5±1.8	38.9±2.3	32.5±5.0
d7 9	1	35.4±22.9	42.9±15.3	12.4±25.9	2.9±2.2	63.8±3.7	20.5±4.6
d7 10	1	39.4±12.8	42.0±8.5	57.3±22.5	24.1±2.8	47.9±2.9	
d14 1	2	66.1±10.4	36.9±4.9	35.6±9.7	45.4±4.8	31.8±2.8	31.1±3.6
d14 2	2	89.4±14.7	22.4±7.6		26.5±4.7		38.6±7.7
d14 3	2	31.7±7.5	35.3±7.9		31.7±3.2	34.5±3.8	27.8±3.9
d14 4	2	28.1±3.7	30.9±7.5		31.4±3.8	33.5±2.9	31.6±4.3
d14 5	2	38.2±7.1	32.3±7.6	31.1±4.4	36.4±3.9	31.3±3.4	28.6±4.1
d14 6	2	33.0±5.2	25.3±7.0		33.9±5.6	31.9±5.0	32.6±4.1
d14 7	2	29.5±6.8	31.5±6.2		41.0±4.6	41.8±2.6	32.9±5.0
d14 8	2	32.8±6.6	32.0±6.0		34.6±4.3	28.3±3.9	28.8±4.5
d17 E1	3	36.1±7.1	32.4±10.4	36.7±6.2	47.5±5.5	42.0±3.5	45.4±4.1
d17 E2	3	33.7±7.6	30.3±5.0	32.2±4.8	37.5±4.1	40.1±5.2	46.0±5.3
d17 E3	3	33.3±5.7	47.9±8.3		61.6±7.8	57.5±5.5	56.8±11.4

d17 E4	3	40.8±4.6	40.6±7.3	31.4±5.0	32.4±7.6	28.1±3.5	26.4±2.7
d17 E5	3	30.7±3.9			32.8±7.6	36.6±4.6	28.3±3.1
d17 E6	3	33.8±6.8	29.4±6.3	34.9±5.6	28.7±4.0	29.0±2.6	23.5±3.7
d17 E7	3	43.4±5.1		33.9±3.5	35.9±4.2	31.0±2.2	30.6±4.2
d17 E8	3	35.1±6.2	35.1±6.5	28.7±3.8	33.5±4.1	30.2±3.0	30.8±4.2
d17 E9	3	37.8±3.9	34.9±5.6	32.0±3.9	34.7±4.2	34.2±2.5	30.9±3.6
d17 E10	3	34.7±4.9	32.5±6.0	33.4±3.6	33.6±4.3	28.0±3.1	34.5±4.7
d17 TP1	4	37.2±6.6	28.3±7.0	31.3±3.8	35.1±4.2	24.1±3.3	23.1±4.4
d17 TP2	4	7.3±5.9	26.6±6.7		26.3±3.3	25.0±2.3	19.8±3.1
d17 TP3	4		32.9±5.8	31.8±3.7	39.3±4.9	25.0±1.9	32.6±4.2
d17 TP4	4			27.5±4.1	33.0±9.5	22.2±2.4	30.8±4.0
d17 TP5	4	36.4±6.6	31.9±6.4	33.8±4.5	38.5±4.5	22.8±2.8	29.1±4.3
d17 TP6	4	36.7±9.3	32.1±7.7	30.1±3.4	34.7±8.8	24.2±3.2	28.6±4.2
d17 TE1	5	33.8±6.5	28.8±6.2	38.5±6.4	30.8±3.8	23.7±2.9	20.3±3.1
d17 TE2	5			24.1±7.8	28.7±3.9	28.0±3.5	19.4±3.7
d17 TE3	5	35.1±5.4	32.3±5.8	33.3±3.8	40.7±5.1	25.2±2.5	26.0±3.8
d17 TE4	5	32.3±5.7		29.2±4.4	34.5±4.2	25.1±3.0	28.4±3.7
d17 TE5	5	36.8±6.3	33.8±9.2		30.3±9.7	24.6±2.7	23.1±3.4
d17 TE6	5	38.9±5.5			30.5±10.2	22.5±2.3	26.1±3.8
d25 1	6	39.2±6.7		33.2±5.7	36.1±4.0	17.0±1.2	29.3±5.1
d25 2	6	39.8±5.6	25.1±6.4	36.5±6.7	36.4±2.7	16.3±0.9	25.8±4.0
d25 3	6	37.4±5.9	35.4±4.5	34.4±4.4	36.0±2.9	15.9±1.5	27.9±4.5
d25 4	6	40.1±4.4	36.8±5.0	32.1±2.9	39.6±2.7	25.9±1.7	29.1±4.5
d25 6	6	39.6±5.9	36.6±4.6	31.5±2.9	38.6±2.2	26.4±1.7	29.6±4.2

d25 7	6	40.6±5.3	40.5±5.0	29.8±2.8	39.3±2.4	25.6±2.1	29.1±4.7
d25 8	6	31.6±3.5	32.8±4.7	30.3±3.6		25.5±2.0	25.1±4.1
d25tro 1	7	33.0±1.8	35.4±4.7	31.1±2.8	40.3±2.8	24.5±1.6	26.8±4.0
d25 tro 2	7	33.3±1.8	34.1±4.3	31.9±3.0	39.9±2.6	23.9±2.1	26.0±4.0
d25 tro 3	7	36.4±8.6	36.1±5.1	32.2±2.9	39.4±2.5	25.5±2.1	26.8±3.9
d25 tro 4	7	37.1±3.3	37.0±4.2		39.4±2.5	25.0±1.9	28.5±4.4
d25 tro 5	7	33.9±1.4	38.3±4.8	32.6±3.0	40.3±2.3	25.5±1.9	29.1±4.5

Individual methylation values for six bovine imprinted gene differentially methylation regions in day 7 blastocysts, day 14 hatched ovoid embryos, day 17 filamentous embryos and day 25 implanting conceptii. ± values represent standard deviation for the average methylation of a given DMR within each sample.
