# Supplementary material for: DNA methylation dynamics at imprinted genes during bovine pre-implantation embryo development
Source: BMC Dev Biol. 2015 Mar 10;15:13. doi: 10.1186/s12861-015-0060-2 (PMC4363183; doi:10.1186/s12861-015-0060-2)
Supplement: Additional file 4: Figure S2A-B. — DNA methylation analysis using limited starting amounts of genomic DNA as input for bisulfite conversions. [file 12861_2015_60_MOESM4_ESM.zip › 12861_2015_60_MOESM2_ESM/2012306920130544_add2c.pdf]

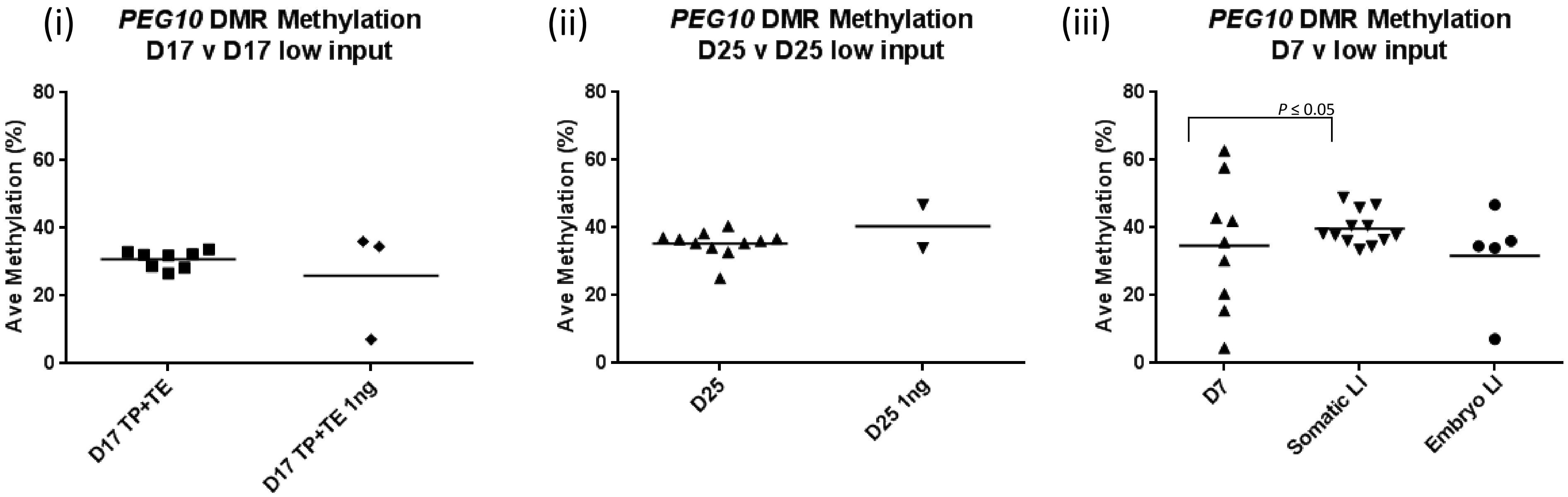

Figure S2C. Analysis of methylation at the *PEG10* DMR using limited starting amounts of DNA. (i) Analysis of D17 TP+TE methylation values (outlined in table S1, *n*=8) vs D17 TP+TE (*n*=3) using 1ng of DNA for bisulfite modification. Methylation was not significantly different. (ii) Analysis of D25 methylation values (table S1, *n*=11) vs D25 (*n*=2) using 1ng of DNA for bisulfite modification showed no significant difference in methylation. (iii) Comparison of D7 values (*n*=9) to low input (LI) somatic DNA (0.5-1ng heart & liver DNA, *n*=12) and LI embryonic DNA (all D17 and D25 1ng samples, *n*=5) showed that D7 variation in methylation was significantly different to somatic LI samples and not with embryo LI samples.
