# Supplementary figures and images for: DNA methylation dynamics at imprinted genes during bovine pre-implantation embryo development
Source: BMC Dev Biol. 2015 Mar 10;15:13. doi: 10.1186/s12861-015-0060-2 (PMC4363183; doi:10.1186/s12861-015-0060-2)

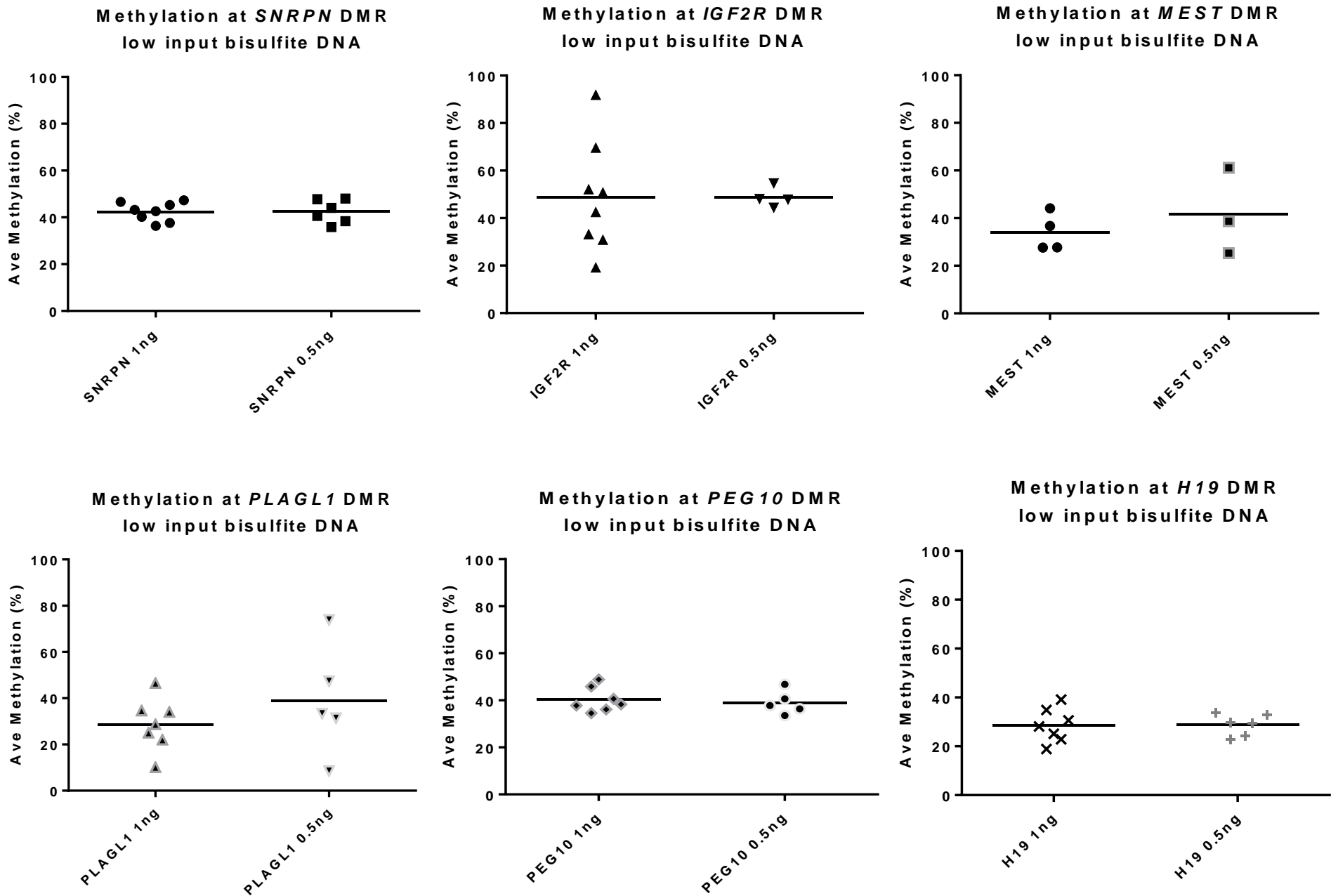

Figure S2A.

Supplement: Additional file 4: Figure S2A-B. — DNA methylation analysis using limited starting amounts of genomic DNA as input for bisulfite conversions. [file 12861_2015_60_MOESM4_ESM.zip › 12861_2015_60_MOESM2_ESM/2012306920130544_add2a.pdf]

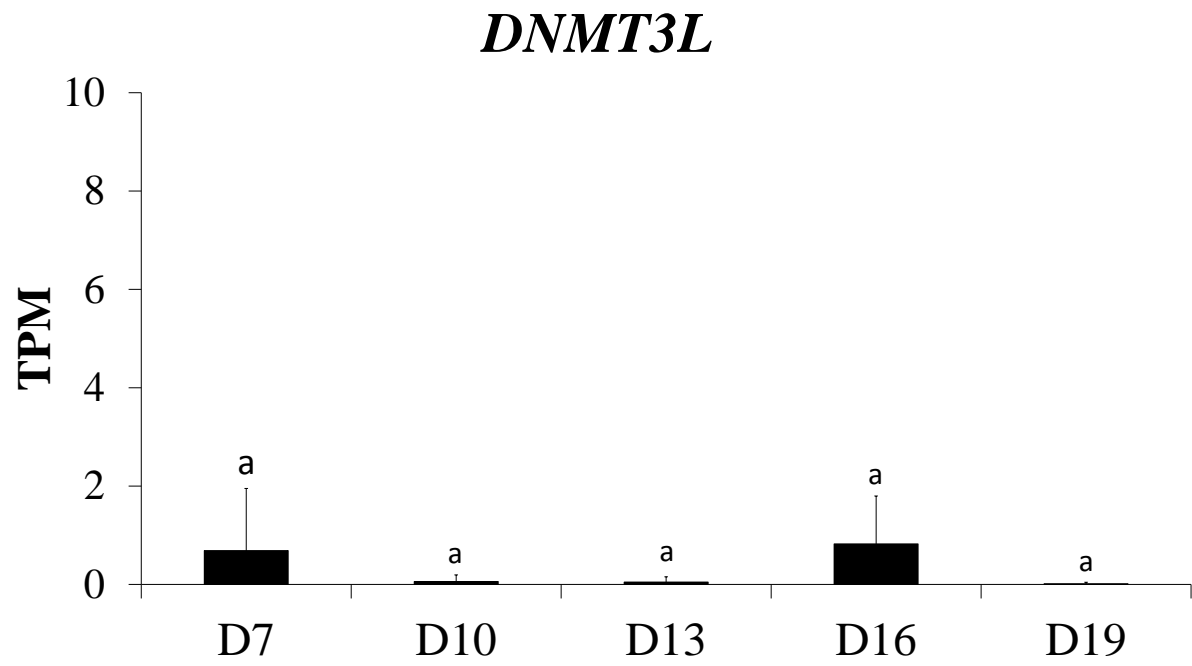

Figure S4. *DNMT3L* mRNA expression during bovine embryonic development.

Supplement: Additional file 5: Figure S4. — DNMT3L mRNA expression during bovine embryonic development. [file 12861_2015_60_MOESM5_ESM.pdf]

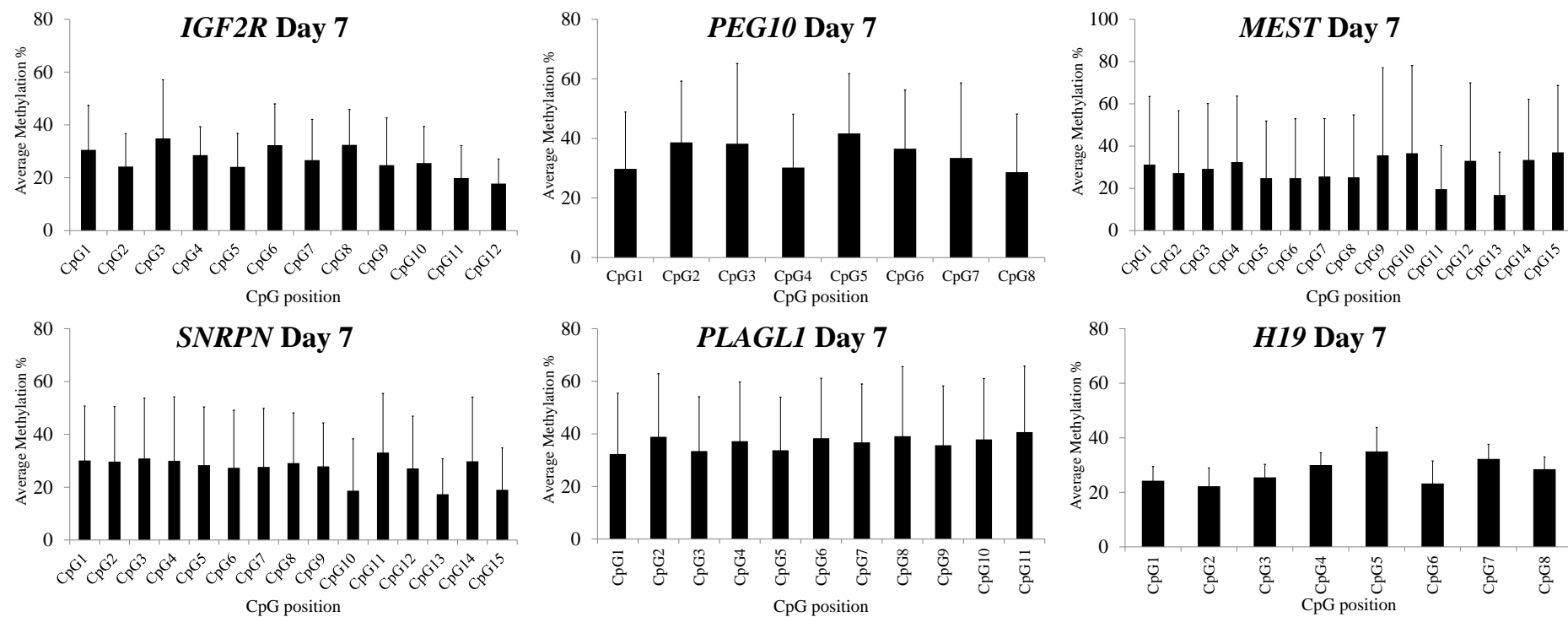

Figure S2. D7 individual CpG methylation analysis.

Supplement: Additional file 6: Figure S3. — D7 individual CpG methylation analysis. [file 12861_2015_60_MOESM6_ESM.pdf]
